# Factors Influencing Outcome After Shoulder Arthroplasty (FINOSA Study): Protocol of a Prospective Longitudinal Study With Randomized Group Allocation

**DOI:** 10.2196/56522

**Published:** 2024-11-18

**Authors:** Anke Claes, Annelien De Mesel, Thomas Struyf, Olivier Verborgt, Filip Struyf

**Affiliations:** 1 Research Group MOVANT, Department of Rehabilitation Sciences and Physiotherapy University of Antwerp Wilrijk Belgium; 2 MORE Foundation Antwerp Orthopedic Center Algemeen Ziekenhuis Monica Deurne Belgium; 3 Academic Centre for General Practice Department of Public Health and Primary Care Katholieke Universiteit Leuven Leuven Belgium; 4 EPI-Centre Department of Public Health and Primary Care Katholieke Universiteit Leuven Leuven Belgium; 5 Department of Orthopaedic Surgery and Traumatology University of Antwerp Campus Drie Eiken Wilrijk Belgium

**Keywords:** shoulder arthroplasty, influencing factors, rehabilitation, arthroplasty, shoulder, FINOSA-study, evidence based, post-operative rehabilitation, rehabilitation protocols, shoulder pain, clinical outcomes, geriatrics, longitudinal study, shoulder dysfunction

## Abstract

**Background:**

There is an increasing need for evidence-based postoperative rehabilitation strategies to optimize patient outcome. Knowledge of potential prognostic factors could steer the development of rehabilitation protocols and could result in better treatment outcomes and higher patient satisfaction.

**Objective:**

This study aimed to investigate which potential prognostic factors predict baseline shoulder pain and function and its evolution in the first 2 years following surgery, in patients with total shoulder arthroplasty. The secondary objective is to investigate which potential prognostic factors predict baseline quality of life and its evolution in the first two years following surgery.

**Methods:**

To reach the aims of this project, a prospective longitudinal study, running from January 2020 to March 2025, will be carried out with a follow-up of 48 months. Patients will be randomized based on sling wear. We will study factors such as shoulder function, patient expectations, psychosocial factors, lifestyle factors, sling wear, soft tissue integrity, and physiotherapy treatment. Test moments will take place preoperatively, at 6 weeks, 12 weeks, 6 months, 12 months, and 24 months. Descriptive statistics will be used to describe the patient population characteristics. Based on literature review, expert opinion, and univariate analyses, potential prognostic factors will be chosen as covariates. A mixed regression model for repeated measures will be used to assess both the evolution of the Shoulder Pain and Disability Index within persons from baseline over time and the differences in evolution between participants. Correlation analyses will be used to investigate associations between the other outcome measures such as the Constant and Murley Score, shoulder range of motion, shoulder muscle strength, and proprioception, and the primary outcome measure, the Shoulder Pain and Disability Index score. Potential prognostic factors not included in the model will be presented in a descriptive manner.

**Results:**

Data collection started in January 2020. In April 2023 the sample size was reached. Data collection will end in April 2025. Analyses will follow when data collection is completed.

**Conclusions:**

Knowledge of potential prognostic factors will have implications toward better rehabilitation strategies of patients after total shoulder arthroplasty.

**Trial Registration:**

ClinicalTrials.gov NCT04258267; https://clinicaltrials.gov/study/NCT04258267

**International Registered Report Identifier (IRRID):**

DERR1-10.2196/56522

## Introduction

Over the last decade, the number of shoulder arthroplasty procedures have more than doubled in the United States [1,2]. Based on registered studies in Australia, the United States, and Europe, the incidence is vastly increasing [1,3-5] with the demand being projected to rise with 755.4% from 2015 to 2030 [6]. Reasons for this large increase in number of shoulder arthroplasty procedures include amongst others, the good clinical outcomes, the expanding indications for reverse shoulder arthroplasty and the growing elderly population [2,7-11].

Due to this substantial boom, there is an increasing need for evidence-based postoperative rehabilitation strategies to optimize patient outcome. However, the consensus in literature is currently lacking. The only evidence-based consensus is that rehabilitation is believed to have an important impact on patient outcomes, and that there is need for high quality prospective longitudinal research [12,13]. Insight in potential prognostic factors, both modifiable and nonmodifiable is needed to improve the outcome of rehabilitation [14]. Modifiable factors are factors that can be influenced by changing the treatment approach, while in nonmodifiable factors, this is not possible. These different factors may both explain the variety of postoperative outcome satisfaction and might play an important role in the rehabilitation after shoulder arthroplasty, for example, age [15-17], gender [15-17], preoperative shoulder function [18,19], patient expectations [20-23], indication for arthroplasty [24], lifestyle factors, psychosocial factors [25,26], patient characteristics [18,23,24,27], and soft tissue integrity [28,29]. However, at this moment it is not clear to which extent these factors have a relevant influence on postoperative outcome [14]. Knowledge of potential prognostic factors could steer the development of rehabilitation protocols and could therefore result in better treatment outcomes and higher patient satisfaction. Studies on (non) modifiable potential prognostic factors for outcome after shoulder arthroplasty are clearly needed [12,14]. Therefore, the objectives of this study are as follows:

- Primary objective: to investigate which potential prognostic factors predict baseline shoulder pain and function measured with Shoulder Pain and Disability Index (SPADI) and the evolution of SPADI in the first 2 years following surgery, in patients with total shoulder arthroplasty.
- Secondary objective: to investigate which potential prognostic factors predict baseline quality of life measured with Short Form 36 (SF-36) and the evolution of quality of life in the first 2 years following surgery, in patients with total shoulder arthroplasty.

We will study factors such as shoulder function, patient expectations, psychosocial factors, lifestyle factors, sling wear, soft tissue integrity, and physiotherapy treatment.

To reach the aims of this project, a prospective longitudinal study will be carried out over 48 months, starting from January 2020 up to March 2025. The reporting of this protocol is in line with the SPIRIT statement recommendations.

## Methods

### Study Setting

Data will be collected monocentric in the shoulder surgery unit of the orthopedic surgery department of AZ Monica in Antwerp, Belgium, starting from January 1, 2020. The estimated ending time of data collection will be 2 years after including the last patient. To standardize the procedure and minimize the effect of different surgeons on outcome, a single fellowship trained shoulder specialized surgeon [OV] will perform all shoulder arthroplasty procedures.

Preoperatively, after being scheduled for a total shoulder arthroplasty, all patients, regardless of the indication for surgery, except for acute fractures, are approached by the principal researcher. The principal researcher will ask the patients to participate right before the surgery. When patients agree to participate, the first measurements will be taken immediately after signing the informed consent. Patients are thoroughly informed about the study before obtaining written consent. The informed consent can be found in Multimedia Appendix 1. The appointments will take place on the following time points: on average 1-2 hours before surgery, at 6 weeks post surgery, at 12 weeks post surgery, at 6 months post surgery, at 12 months post surgery, and 24 months post surgery.

All patients will undergo the same testing procedure. Measurements will be carried out by 2 researchers [AC and ADM]. Each researcher was trained in performing these measurements. Parallel to this study, a reliability study regarding these measurements is ongoing. To ensure adequate follow-up, the consultation schedule of the orthopedic surgeon will be followed. At every time point, patients plan the next consultation with the orthopedic surgeon.

### Eligibility Criteria

The population of interest includes patients undergoing an anatomical or reverse total shoulder arthroplasty, both from traumatic and nontraumatic origin. Inclusion criteria are adult men and women scheduled for a primary total shoulder arthroplasty. Exclusion criteria are patients scheduled for revision surgery, acute fractures, patients not understanding the Dutch language, surgeries where perioperative complications occur, and patients that cannot be randomized.

### Surgical Intervention

The deltopectoral approach will be used in all patients. If applicable, hardware from previous surgery will be removed and a resection of the subdeltoid adhesions will be done. In all cases, a tenodesis of the long head of the biceps will be performed just above the upper border of the pectoralis major tendon using a suturing technique. A tenotomy of the subscapularis tendon will be performed 1 cm from its insertion on the minor tuberosity. In stiff shoulders, the subscapularis tendon is peeled-off starting more lateral at the bicipital groove. Both subscapularis tendon and capsule will be released toward medial, and a resection of the contracted anterior capsule will be performed. After dislocation of the humeral head, resection of osteophytes on the humeral head will be done. For anatomic total shoulder arthroplasty, a resection of the humeral head with an extramedullary guide will be performed at the anatomic neck following the anatomical version and inclination. For reverse total shoulder arthroplasty, a resection of the humeral head at 145° inclination and 10° retroversion is performed with the use of an intramedullary guide.

After exposure of the glenoid surface, labrum and osteophytes of the glenoid are removed and any remaining cartilage on the articular surface of the glenoid is removed. In anatomic total shoulder arthroplasty, the glenoid surface is reamed and a hybrid anatomical glenoid (combination of central ingrowth post and cemented peripheral pegs) is then placed in the preoperatively planned position. For reverse total shoulder arthroplasty, a standard baseplate with central post and 2 peripheral screws is positioned in the preoperatively planned position and a glenosphere is placed. Then the humeral side is finished. For anatomic total shoulder arthroplasty, a stemless, uncemented component is positioned using an anatomically sized humeral head. For reverse total shoulder arthroplasty, the proximal humeral canal is prepared for a stemmed, uncemented humeral component. After reduction the subscapularis tendon is anatomically repaired using a transosseus suturing technique in anatomic total shoulder arthroplasty. In reverse total shoulder arthroplasty, the subscapularis is only repaired if reduction of the tendon is possible in 30° of external rotation and the posterior remnants of the rotator cuff are intact.

### Outcome Measures

We selected the outcome measures based on the available literature and clinical expertise. Shoulder pain and shoulder function will be measured using the SPADI, which is the primary outcome measure. Quality of life measured with the SF-36, is the secondary outcome measure. Other measured outcomes are shoulder pain and function measured with the Constant and Murley Score (CS), the Visual Analogue Scale (VAS), active shoulder range of motion, shoulder muscle strength, and proprioception of the shoulder. The last outcome measure is patient satisfaction, measured with a self-developed questionnaire.

To measure shoulder pain and function, the SPADI [30] has shown a good internal consistency and test-retest reliability, with a Cronbach α of 0.94 and Intraclass Correlation Coefficient of 0.89 (95% CI 0.83-0.93). The questionnaire is divided in 2 subscales, a pain subscale with 5 statements, and a disability subscale with 8 statements. Every statement must be scored with a number between 0 and 10, with 0 being “no pain” or “no effort” and 10 being “the worst pain” or “so difficult that help is needed”. The overall SPADI score will be used and is calculated: ([sum of all statement scores]/130)×100. This self-administered questionnaire is available in Dutch, and is designed to measure pain and disability associated with shoulder pathology in different clinical settings [31].

Shoulder pain will be measured with the VAS (0-100). Patients will be asked to score the average pain they experience during rest and during shoulder movement on a drawn line of 100 mm, with answers ranging for 0-100. With 0 showing “no pain” and 100 showing “the worst pain.” Shoulder pain and function will be measured with the CS, validated and reliable in patients with shoulder dysfunction [32,33]. We used a Dutch translation, which is not yet validated. A score will be calculated between 0-100, with 100 being a more functional patient.

To measure the active shoulder range of motion, 7 active range of motion measurements of the shoulder will be measured (Table 1). All measurements will be performed in sitting position to avoid compensatory movement of the trunk. A gravity inclinometer will be used during this procedure (Plurimeter, Dr Rippstein). First, anteflexion, abduction, and abduction in the scapular plane will be measured [34]. These measurements are expressed in degrees. After that, both internal rotation and external rotation will be measured. The participant must reach behind his back and is asked to try to go as far up as possible with his thumb to measure functional glenohumeral internal rotation. The position of the thumb will be noted following an ordinal scale (Table 1) [34]. To measure the functional glenohumeral external rotation, the participant is asked to try and put his hand in his neck. The position of the hand will be noted following an ordinal scale (Table 1) [32]. At last, internal rotation and external rotation glenohumeral range of motion will be measured in the horizontal plane. The participant rotates the arm upward and downward, performing a glenohumeral internal and external rotation. Both measurements will be measured with the inclinometer and expressed in degrees.

To measure the shoulder muscle strength, 5 different muscle strength measurements of the shoulder will be performed in sitting position to avoid compensatory movement of the trunk (Table 2). A handheld dynamometer (HHD, Microfet) is used. During all muscle strength measurements, the participant is asked to hold the position while the rater applies a force with the HHD. The rater’s force will be increased until the participant’s arm moves, or the participant indicates the maximal force is reached. During muscle strength measurement of internal and external rotation, the rater can provide stabilization at the distal end of the humerus with the nontesting hand. It is important that the participant does not move his or her elbow medially of laterally. All these muscle strength measurements are expressed in Newton.

The joint positioning sense test and force sensation test measured participant’s ability to actively reproduce an active positioning of the arm and to actively reproduce target forces with shoulder muscle contraction, respectively. To measure the proprioception, the joint positioning sense and force sensation will be measured (Table 3). First, the joint positioning sense will be measured in 4 different angles. The participant is asked to engage a movement of the shoulder in his or her own tempo with eyes open. The rater says “stop” when the shoulder is in the previous decided position (criterion angle), and the participant holds this for 3 seconds to become aware of the position. The participant will then be asked to return to the starting position and to close his or her eyes. Then the participant is asked to reproduce the same position or angle (reproduced angle) with his or her eyes closed for 3 seconds and to give a sign when he or she thinks the angle was reached. This will be repeated 2 times. The degrees will be noted and the absolute difference between criterion and reproduced angle (reproducing error) will be taken as measure for proprioceptive accuracy [35]. To measure the force sensation for internal and external rotation, the HHD will be used. To begin the force sensation measurement the participant is asked to produce 50% of the maximally voluntary contraction, calculated from the muscle strength test (see above), while receiving visual and verbal feedback regarding the force being produced. When the participant reaches the target force, he or she will be asked to hold it for 3 seconds to become aware of the force. After 3 seconds, the participant is instructed to relax and to close their eyes so the visual feedback would be removed. Then the participant is asked to reproduce the force. The participant will be asked to verbally indicate when he or she reaches the target force. This will be repeated 2 times and the reproducing error in degrees will be calculated by the absolute difference between criterion force and reproduced force, which is taken as measure for proprioceptive accuracy [36].

Patient satisfaction will be measured with a self-developed questionnaire, adapted from the study of Swarup et al [22]. This questionnaire consists of 8 questions in Dutch, regarding the satisfaction of the patient in terms of pain, function, and quality of life.

Quality of life will be measured with the SF-36 [37,38]. This is a self-administered validated survey of patient health, comprising mental health as well as physical health, available in Dutch [39].

**Table 1:** Active shoulder range of motion measurements.

Range of motion measurement	Position participant	Position hand of participant	Position inclinometer	Unit
Glenohumeral anteflexion	Sitting	Pointing upward to ensure consistent rotation	Ventral on distal humerus, perpendicular to the plane of motion	Degrees
Glenohumeral abduction	Sitting	Pointing upward to ensure consistent rotation	Lateral on distal humerus, perpendicular to the plane of motion	Degrees
Glenohumeral abduction scapular plane	Sitting	Pointing forward to ensure consistent rotation	Ventral on distal humerus, perpendicular to the plane of motion	Degrees
Functional internal rotation	Sitting	—^a^	No inclinometer used	Ordinal scale: hand on the lateral side of the major trochanter, thumb behind buttocks, thumb toward contralateral SI joint, thumb toward L3, thumb toward T12, or thumb between scapulae^b^
Functional external rotation	Sitting	—^a^	No inclinometer used	Ordinal scale: hand not in neck or hand only to mouth, hand in neck but elbow pointing forward, hand in neck and elbow ½ open, hand in neck and elbow completely open, hand in neck and patient can extend elbow to 90° of flexion (hooray), and hand in neck and patient can extend the arm to complete elevation^c^
Internal rotation in horizontal plane	Sitting	Elbow in 90° of flexion, palm of the hand downwards	Middle of dorsal forearm	Degrees
External rotation in horizontal plane	Sitting	Elbow in 90° of flexion, palm of the hand downwards	Middle of dorsal forearm	Degrees

^a^—: not applicable.

^b^See Multimedia Appendix 2 for visual representation of the ordinal scale for functional internal rotation.

^c^See Multimedia Appendix 3 for visual representation of the ordinal scale for functional external rotation.

**Table 2:** Shoulder muscle strength measurements.

Isometric muscle strength measurement	Position participant	Position hand of participant	Position HHD^a^	Direction of applied force (HHD^a^)	Unit
Anteflexion 90°	Sitting, shoulder in 90° of anteflexion, and 30° in frontal plane toward abduction, arm extended	Palm of the hand downwards	Dorsal on distal forearm	Downward orientated	Newton
Anteflexion	Sitting, arm by the side of the trunk with elbow 90° of flexion	Thumb pointing upward to ensure consistent rotation	Ventral on humerus	Posterior orientated	Newton
Abduction	Sitting, arm by the side of the trunk with elbow 90° of flexion	Thumb pointing upward to ensure consistent rotation	Lateral on humerus	Medial orientated	Newton
Internal rotation	Sitting, arm by the side of the trunk with elbow 90° of flexion	Thumb pointing upward to ensure consistent rotation	Medial on distal forearm	Lateral orientated	Newton
External rotation	Sitting, arm by the side of the trunk with elbow 90° of flexion	Thumb pointing upward to ensure consistent rotation	Lateral on distal forearm	Medial orientated	Newton

^a^HHD: handheld dynamometer.

**Table 3:** Proprioception measurements.

Proprioception measurement	Position participant	Position of hand	Position inclinometer or HHD^a^	Unit
Joint positioning sense test 45° ± 10° anteflexion	Standing with elbow extended	Thumb pointing upward to ensure consistent rotation	Attached on ventral side distal humerus	Repositioning error in degrees
Joint positioning sense test 90° ± 10° anteflexion	Standing with elbow extended	Thumb pointing upward to ensure consistent rotation	Attached on ventral side distal humerus	Repositioning error in degrees
Joint positioning sense test 45° ± 10° abduction	Standing with elbow extended	Thumb pointing forward to ensure consistent rotation	Attached on lateral side distal humerus	Repositioning error in degrees
Joint positioning sense test 90° ± 10° abduction	Standing with elbow extended	Thumb pointing forward to ensure consistent rotation	Attached on lateral side distal humerus	Repositioning error in degrees
force sensation test internal rotation (50% MIVC)	Sitting, arm by the side of the trunk with elbow 90° of flexion	Thumb pointing upward to ensure consistent rotation	Medial on distal forearm, force lateral orientated	Reproducing error in Newton
force sensation test external rotation (50% maximally voluntary contraction)	Sitting, arm by the side of the trunk with elbow 90° of flexion	Thumb pointing upward to ensure consistent rotation	Lateral on distal forearm, force medial orientated	Reproducing error in Newton

^a^HHD: handheld dynamometer.

### Patient Characteristics

Age, gender, comorbidities, previous surgery, and indication for surgery are collected from the medical records of the patient. Body weight and length will be asked during every consultation.

### Potential Prognostic Factors

Patient characteristics such as body weight and body length, to calculate the BMI. Preoperative shoulder function including shoulder range of motion, shoulder muscle strength, and proprioception of the shoulder before surgery (Tables 1-3).

Soft tissue integrity of the different muscles around the shoulder joint will be collected from the surgeon’s report and from previous surgeries. The shoulder muscles of interest are the rotator cuff muscles, biceps brachii muscles, and triceps brachii muscles. Soft tissue will be scored a 1 if it is damaged either through injury of previous surgery (open or arthroscopic surgery). Soft tissue will be scored 0 if it is not damaged, and no previous surgery has occurred.

Patients’ expectations will be measured with a self-developed questionnaire, based on the HSS Shoulder Surgery Expectations Survey [20]. This survey consists of 17 questions, in Dutch, regarding postoperative expectations of symptom relief, physical function, and psychosocial function. Both spectrum and importance of expectations are measured, in less than 5 minutes. Each question has the following 5 possible responses: “very important,” “somewhat important” “a little important,” “I do not expect this,” and “this does not apply to me.”

Sling wear is also a possible prognostic factor. Patients will be randomized preoperatively in 2 groups with varying immobilization period. Patients in the first group (delayed mobilization group) will be wearing a sling for 6 weeks, the first 4 weeks an abduction sling, followed by 2 weeks of adduction sling. Patients in the second group (early mobilization group) will be wearing an adduction sling for 4 weeks. If a patient cannot be randomized in one of the 2 groups, the patient will be excluded from the study. For example, if a longer immobilization period is needed (complications during surgery, bone grafts used, etc.). Raters will be blinded from the sling wear throughout the whole observation period.

Psychosocial factors**,** including anxiety, depression, pain catastrophizing, and self-efficacy will be measured. Anxiety and depression will be measured by the Hospital Anxiety and Depression Scale [40]. This is a validated, self-administered questionnaire, available in Dutch, measuring anxiety as well as depression using 14 items. The items are scored on a 4-point Likert scale (0-3), the total score ranges from 0-21, whereas 0 means no depression or anxiety disorder and 21 means probability for anxiety or depression disorder, with a cut-off of +8 [41,42]. Pain catastrophizing will be measured with the Pain Catastrophizing Scale [43], a validated for low back pain, self-reported questionnaire that can be completed and scored within 5 minutes, available in Dutch [44,45]. This questionnaire consists of 13 items in which patients are asked to reflect on painful experiences and indicate feelings and thoughts about this pain. The questions are scored on a 5-point Likert scale, ranging from 0 (not at all) to 4 (always). Self-efficacy will be measured by the Pain Self-Efficacy Questionnaire, a validated, self-administered questionnaire with an excellent internal consistency, available in Dutch [46,47]. The questionnaire consists of 10 items whose are scored on a 7-point Likert scale, depending on how confident the patient is about a particular activity, despite the pain, 0 being not confident at all and 6 being completely confident. A total score will be calculated ranging from 0 to 60, with 60 indicating greater levels of confidence dealing with pain.

Lifestyle factors, including physical activity, smoking behavior, sleep, and drug and alcohol usage. Physical activity will be measured by the International Physical Activity Questionnaire *– long version* [48]. This is a valid and reliable questionnaire, available in Dutch [49]. Smoking behavior, sleep, and drug and alcohol usage will be objectified using a self-developed questionnaire. Drug usage will also be collected from the medical record. An overview of measurements per time point can be found in Table 4.

Physiotherapy treatment will also be objectified. Physiotherapists treating the patients included will be sent a questionnaire. The number of physiotherapy sessions during the first 6 weeks will be listed, as well as the number of given home exercises. The ratio between these 2 numbers indicates whether patients did perform predominantly more physiotherapy sessions or more home exercises. All patients will get the same information regarding postoperative rehabilitation. The first 2 weeks, a home-exercise program will be followed, explained by the inpatient physiotherapist. After 2 weeks the patient will be asked to visit the outpatient physiotherapist of their choice. An overview of measurements per time point can be found in Table 4.

**Table 4:** Overview of measurements per time point.

Measurements		Type	T1 (preoperative)		T2 (6 weeks postoperative)		T3 (12 weeks postoperative)		T4 (6 months postoperative)	T5 (12 months postoperative)		T6 (24 months postoperative)
**Patient characteristics**												
Age	Medical records		X	—^a^		—^a^		—^a^		—^a^	—^a^	
Gender	Medical records		X	—^a^		—^a^		—^a^		—^a^	—^a^	
Comorbidities	Medical records		X	—^a^		—^a^		—^a^		—^a^	—^a^	
Previous shoulder surgery	Medical records		X	—^a^		—^a^		—^a^		—^a^	—^a^	
Indication for surgery	Medical records		X	—^a^		—^a^		—^a^		—^a^	—^a^	
**Outcome measures**												
Shoulder function	SPADI^b^ VAS^c^ CS^d^ ROM Strength Proprioception		X X X X X X	X X X X —^a^ —^a^		X X X X X X		X X X X X X		X X X X X X	X X X X X X	
Patient satisfaction	Self developed questionnaire		—^a^	X		X		X		X	X	
Quality of Life	SF-36^b^		X	X		X		X		X	X	
**Potential prognostic factors**												
Patient characteristics	Body weight Body length		X X	X X		X X		X X		X X	X X	
Preoperative shoulder function	ROM Strength Proprioception		X X X	X —^a^ —^a^		X X X		X X X		X X X	X X X	
Patient expectations	Self developed questionnaire		X	—^a^		—^a^		—^a^		—^a^	—^a^	
Soft tissue integrity	Surgery report		—^a^	—^a^		—^a^		—^a^		—^a^	—^a^	
Sling wear	Randomization		X	—^a^		—^a^		—^a^		—^a^	—^a^	
Psychosocial factors	Anxiety and depression (Hospital Anxiety and Depression Scale) Self-efficacy (Pain Self-Efficacy Questionnaire) Catastophizing (Pain Catastrophizing Scale)		X X X	X X X		X X X		X X X		X X X	X X X	
Lifestyle factors	Drug usage Physical activity (International Physical Activity Questionnaire – long version) Smoking behavior Sleep Alcohol use		X X X X X	X X X X X		X X X X X		X X X X X		X X X X X	X X X X X	
Physiotherapy treatment	Questionnaire		X	X		X		X		X	X	

^a^—: not applicable.

^b^outcomes used in the mixed-models analysis.

^c^VAS: Visual Analogue Scale.

^d^CS: Constant and Murley Score.

### Participant Timeline

**Figure 1:**
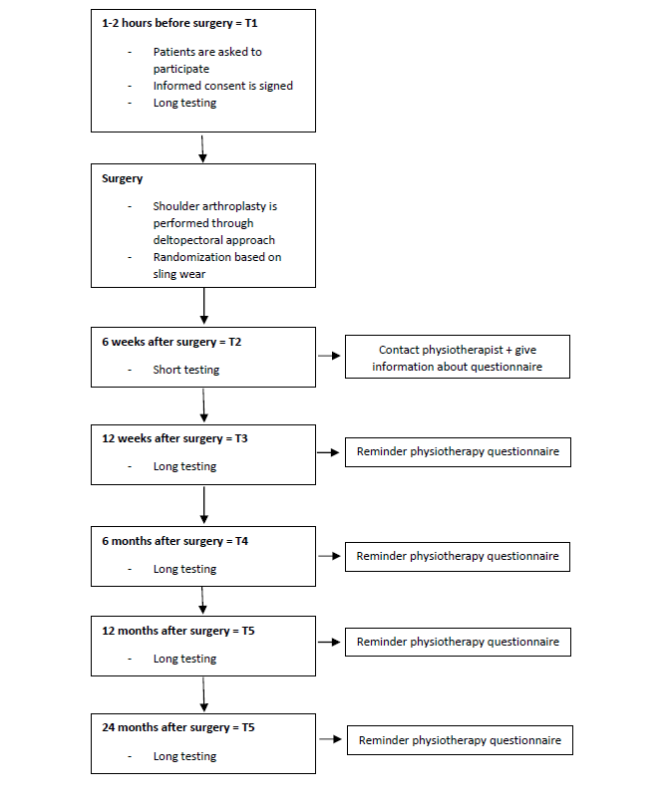
Participant timeline.

The participant timeline is shown in Figure 1.

### Sample Size

Considering a mixed regression model for repeated measures and using a moderate effect size that is still clinically relevant (20 points average decrease in SPADI score), inclusion of 5 covariates, confidence level (α=0.05), and desired power (90 %), the required total sample size is calculated to be 43 participants per group (Edland method, R package longpower 1.0-11) [50-52]. The SD of the SPADI measurements is set at 18, and is estimated from the SD measured in previous studies [31]. The variance of the residuals cannot be estimated from previous studies, but this variance only has a very limited effect on the sample size. It doesn’t change the result of the power calculation and is not included in this analysis. Given the expected dropout rate of approximately 12.5%, a total number of 97 patients will be needed. The power is set at 90% to minimize the chance of making a type II error.

### Statistical Methods

Statistical analyses will be performed using SAS Studio (version 9.4; SAS Institute Inc). Level of significance is set at *P*=.05. Descriptive statistics will be used to describe the patient population. Based on literature review, expert opinion and univariate analyses potential prognostic factors will be chosen as covariates. A mixed regression model for repeated measures will be used to assess both the evolution of the SPADI score and the SF-36 score within persons from baseline over time and the differences in evolution between participants. This type of model has the advantage that the assumption of sphericity can be relaxed. Sphericity will probably be violated, as both the SPADI score, and SF-36 score at baseline and at all the different time points will probably have different variances. Second, using mixed models it is not necessary to have complete datasets to produce accurate results. Multiple imputation of missing values is thus not needed. All available data of a participant can be used in the model estimation, even if people missed a visit. Another advantage is that it is possible to simultaneously examine within and between person phenomena that might contribute to change in outcome. A fourth advantage is that the correlation between observations within persons over time is adequately addressed [53]. One assumption that must be checked is normality of the residuals, using a Shapiro-Wilk test. If these residuals are not normally distributed, an appropriate transformation will be performed first, or a nonlinear mixed model will be used.

Correlation analyses will be used to search for correlations between the other outcomes measured such as CS, shoulder range of motion, shoulder muscle strength, and proprioception and the primary outcome measure, SPADI. Potential prognostic factors not included in the model will be described.

### Data Management

Two separated and secured computers will be used. Patients will be given a specific number characteristic. The respective number-patient relation will be stored on another external hard drive remote from the data computer.

The primary investigator [AC] will perform all data analysis. An independent statistician [TS] will guide the primary investigator in performing statistics.

### Ethical Considerations

The central and local ethical committees of the University Hospital Antwerp and AZ Monica approved this study (B300201942512; 19/48/559). The present study underlies the principles of the Helsinki Declaration. Only data of patients who gave informed written consent to the project will be considered for analysis. The coding list of target data will be saved in a secured folder on an external hard drive. Only the project leader, study nurse, principal investigator, and second investigator have access to it. Between the members of the research team only coded and deidentified data will be shared.

The research team is committed to full disclosure of the results of the study. The results of the study will be disseminated for research purpose at different conferences and as published articles in peer reviewed journals. Findings will be reported in accordance with international guidelines, and we aim to publish in high impact journals. Given the multitude of outcome parameters, results will be divided over several papers.

## Results

Data collection started in January 2020. In April 2023, the sample size was reached. Data collection will end in April 2025.

**Figure 2:**
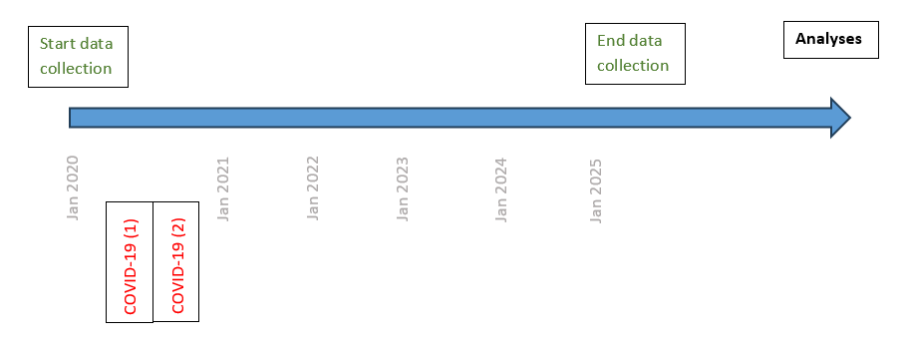
Timeline of study flow.

Due to the COVID-19 pandemic, the duration to reach the sample size was rather long. Between March 2020 and July 2020, and between October 2020 and December 2020, all nonurgent surgeries were postponed and even consultations were cancelled. Therefore, no new patients could be included, and the follow-up of some patients could not be reassured. An overview of the timeline can be found in Figure 2.

## Discussion

### Principal Findings

The aim of this study is to investigate which prognostic factors could possibly predict baseline shoulder pain and function, and quality of life, and the evolution of shoulder pain and function, and quality of life in the first 2 years after a total shoulder arthroplasty surgery. A broad spectrum of factors is studied. This is the first longitudinal study to investigate the possible prognostic factors in patients with a shoulder arthroplasty with adequate power. The longitudinal design will allow us to follow patients over 24 months, which makes it possible to analyze and compare the measurements at 6 time points. Only 1 orthopedic surgeon performed the surgeries. This means the surgical technique is not one of the factors that must be taken into account. Parts of the surgery that do differ between patients are taken into account, such as subscapularis handling, and take-down and repair of muscles. The surgery, preoperative screening, postoperative consultation schedule, and everyday practice is the same in each of the included patients. The questionnaires the HSS Shoulder Surgery Expectations Survey and a self-developed questionnaire, adapted from the study of Swarup et al [22] were translated to Dutch for the purpose of this study. Therefore, their validity in the Dutch-speaking population has yet to be established.

As previously mentioned, the amount of shoulder arthroplasty procedures is rising. In contrast with knee and hip arthroplasties, more follow-up consultations with the orthopaedic surgeon are planned with total shoulder arthroplasties. Patients return to regular consultations at the defined timepoints which minimizes the loss to follow-up. Knowledge of potential prognostic factors will have implications toward better rehabilitation strategies of patients after total shoulder arthroplasty**.** With the modifiable potential prognostic factors analyzed, we could change strategies to try and change the outcome. Also, nonmodifiable factors are of interest. We could educate these patients to change or monitor the patient expectations to increase the quality of the outcomes.

## Appendix

Multimedia Appendix 1 Informed consent.

Multimedia Appendix 2 Visual representation of the ordinal scale for functional internal rotation.

Multimedia Appendix 3 Visual representation of the ordinal scale for functional external rotation.

## Abbreviations

CS: Constant and Murley Score
HHD: handheld dynamometer
SPADI: Shoulder Pain and Disability Index
SF-36: Short-Form 36
VAS: Visual Analogue Scale

## Data Availability

The datasets generated during this study will be made available after analyses from the corresponding author on reasonable request.
