# Supplementary material for: Factors Influencing Outcome After Shoulder Arthroplasty (FINOSA Study): Protocol of a Prospective Longitudinal Study With Randomized Group Allocation
Source: JMIR Res Protoc. 2024 Nov 18;13:e56522. doi: 10.2196/56522 (PMC11612598; doi:10.2196/56522)
Supplement: Multimedia Appendix 1 [file resprot_v13i1e56522_app1.docx]

**Informatiebrochure voor de patiënt**

**Welke factoren hebben een invloed op de uitkomst na een schouder prothese? Een prospectief longitudinaal onderzoek met gerandomiseerde groep toekenning: Op weg naar betere revalidatie strategieën.**

Gelieve onderstaande informatie te lezen om u te helpen bij het beslissen of u al dan niet aan deze studie wenst deel te nemen. Indien er vragen zijn omtrent deze brochure kan u steeds de persoon weergegeven onderaan deze brochure contacteren. Als u beslist om deel te nemen aan deze studie, zal u gevraagd worden een "Toestemmingsformulier" (bijgevoegd bij deze informatiebrochure) te ondertekenen. U houdt deze informatiebrochure best bij zodat u deze in de toekomst nog kunt raadplegen.

**Doel van de studie**

Schouderprothesen worden meer en meer geplaatst. Het is aangetoond dat de revalidatie na de operatie heel belangrijk is bij patiënten met een schouderprothese, maar het is niet duidelijk hoe deze er concreet moet uitzien. Het betreffende project zal onderzoeken welke factoren tijdens de revalidatie hierop een invloed kunnen hebben en hoe we hierop zouden kunnen ingrijpen.

**Praktisch**

U bent gecontacteerd omdat u binnenkort een schouderprothese zal krijgen. Bij deelname aan deze studie, zal u worden ingedeeld in een van de twee studiegroepen. Een groep zal na de operatie vier weken een sling (draagdoek om uw arm en nek ter ondersteuning van uw schouder) dragen, de andere groep zal zes weken een sling dragen. Verder zal u de revalidatie doorlopen onder begeleiding van uw kinesitherapeut naar uw voorkeur. Telkens u op controle komt zullen er enkelen testen en vragenlijsten worden afgenomen. Deze testmomenten zullen doorgaan op de volgende tijdstippen: (1) Voor de operatie (2) zes weken na de operatie, (3) 12 weken na de operatie, (4) zes maanden na de operatie, (5) 12 maanden na de operatie, en (6) 24 maanden na de operatie. De tests zullen bestaan uit het invullen van vragenlijsten omtrent de functie van uw schouder, pijn, kwaliteit van leven, psychosociale factoren, fysieke activiteit en levensstijl en het meten van je beweeglijkheid, spierkracht en proprioceptie rondom de schouder. Dit alles zal in totaal ongeveer 1 uur en 15 minuten van uw tijd innemen. Aan het begin van de studie zal via uw medisch dossier de volgende informatie verzameld worden: leeftijd, geslacht, staat van uw schouderspieren (na de operatie), eventuele aandoeningen, gebruik van medicatie en de resultaten van de online vragenlijst. Op de verschillende testmomenten zullen we eveneens volgende informatie verzamelen via uw medisch dossier: de resultaten van de online vragenlijst.

**Uw risico's**

Er zijn geen risico’s voor deelname aan dit onderzoek.

**Verzekering van de confidentialiteit**

Uw privacy wordt beschermd in de studie. Alle persoonlijke informatie die verworven wordt, blijft strikt vertrouwelijk. Alle gegevens van uw onderzoek worden gepseudonimiseerd. Dit wil zeggen dat alle namen vervangen worden door een codenummer zonder persoonlijke informatie zoals uw naam, adres, of telefoonnummer, zodat uw persoonlijke identiteit onbekend blijft voor de uitvoerende onderzoeker. Deze gegevens worden bewaard door de verantwoordelijke van deze studie – ondergetekende, Prof. dr. Filip Struyf. Op basis van de privacywetgeving hebt u het recht op inzage in de gegevens die over u verzameld werden en het recht om deze gegevens te laten corrigeren indien nodig. De studie werd goedgekeurd door de Commissie Medische Ethiek van het Universitair Ziekenhuis Antwerpen en AZ Monica en alle proefpersonen zijn tijdens de studie verzekerd. Uw deelname aan de studie is vrijwillig en u kunt de studie stoppen op elk moment. Dit zal geen effect hebben op uw medische zorg. Indien u akkoord gaat om aan deze studie deel te nemen, zal u gevraagd worden het “Toestemmingsformulier” te ondertekenen.

**Kosten / Vergoedingen**

Er zijn geen kosten voor u verbonden aan de deelname aan de studie.

**Vertrouwelijkheid**

De informatie die verzameld wordt tijdens deze studie is vertrouwelijk. Uw naam zal niet vermeld zijn op de rapporten en publicaties die voortvloeien uit deze studie. Ter bescherming van de persoonlijke data en bescherming tegen onbevoegde toegang, zullen de resultaten gepseudonimiseerd worden en zullen deze vertrouwelijk worden behandeld, dit in overeenstemming met de Belgische Wetgeving voor de Bescherming Persoonsgegevens. De Universiteit Antwerpen is als opdrachtgever van de studie verantwoordelijk voor de verwerking van uw persoonsgegevens. Zij heeft daartoe een functionaris voor de gegevensverwerking aangesteld. Alle gegevens worden 20 jaar bewaard na het aflopen van het onderzoek. De gegevens worden opgeslagen op een externe harde schijf die na het opladen van de gegevens op een veilige plaats bewaard wordt, waartoe enkel de onderzoeker toegang heeft.

Op de studiedocumenten zal u geïdentificeerd worden d.m.v. een uniek patiënt identificatienummer, uw initialen en uw geboortedatum. Indien u vragen hebt, aarzel niet om een van onderstaande personen te contacteren.

Prof. dr. F. Struyf – [filip.struyf@uantwerpen.be](mailto:filip.struyf@uantwerpen.be)

**Revalidatiewetenschappen en Kinesitherapie**

Campus Drie Eiken – Lokaal R3.09

Universiteitsplein 1, Wilrijk, België4

Prof. dr. I. Baert – [Isabel.baert@uantwerpen.be](mailto:Isabel.baert@uantwerpen.be)

**Revalidatiewetenschappen en Kinesitherapie**

Campus Drie Eiken – Lokaal R3.09

Universiteitsplein 1, Wilrijk, België

Prof. dr. O. Verborgt – [olivier.verborgt@outlook.com](mailto:olivier.verborgt@outlook.com)

**Antwerp Orthopedic Center**

AZ Monica – Florent Pauwelslei 1

2100 Deurne, België

Dra. Anke Claes - [anke.claes@uantwerpen.be](mailto:anke.claes@uantwerpen.be)

|  | **Revalidatiewetenschappen en Kinesitherapie**  Campus Drie Eiken - Lokaal R3.14  Universiteitsplein 1 - 2610 Wilrijk – België |
| --- | --- |

Mevr. A. De Mesel - [annelien](mailto:filip.struyf@uantwerpen.be)@rehab-training.be

|  | **MORE Foundation**  AZ Monica – Florent Pauwelslei 1  2100 Deurne – België |
| --- | --- |

| 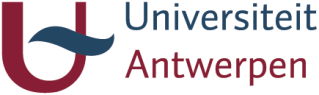 | | |
| --- | --- | --- |
|  | | **Revalidatiewetenschappen en Kinesitherapie** |
|  | |  |
|  | [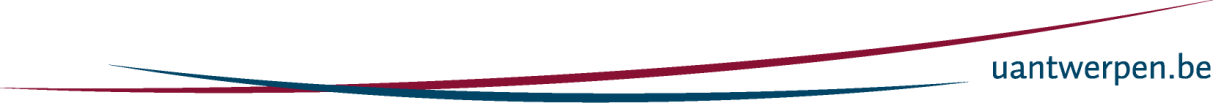](https://www.uantwerpen.be/) | |

**TOESTEMMINGSFORMULIER (Informed Consent)**

Titel van de studie:

**Welke factoren hebben een invloed op de uitkomst na een schouder prothese? Een prospectief longitudinaal onderzoek met gerandomiseerde groep toekenning: Op weg naar betere revalidatie strategieën.**

**INSTRUCTIES VOOR DE PATIENT: gelieve deze vragenlijst zelf in te vullen. Omcirkel het juiste antwoord**

Hebt U de informatiebrochure voor de patiënt gelezen? JA/NEEN

Hebt U de mogelijkheid gehad om vragen te stellen over de studie? JA/NEEN

Hebt U genoeg antwoorden gekregen op al Uw vragen? JA/NEEN

Hebt U genoeg informatie ontvangen over de studie? JA/NEEN

Begrijpt U dat U vrij bent om de studie stop te zetten:

* op elk moment

* zonder verantwoording te moeten geven voor de stopzetting

* en zonder dat dit Uw verdere medische opvolging zal beïnvloeden? JA/NEEN

Gaat U akkoord om deel te nemen aan deze studie? JA/NEEN

Handtekening van de patiënt: ______________________________

Datum: ____ / ____ / _____

Naam van de patiënt: ______________________________

Handtekening van de onderzoeker: ______________________________

Datum: ____ / ____ / _____

Naam van de onderzoeker: _____
