# Supplementary figures and images for: Factors Influencing Outcome After Shoulder Arthroplasty (FINOSA Study): Protocol of a Prospective Longitudinal Study With Randomized Group Allocation
Source: JMIR Res Protoc. 2024 Nov 18;13:e56522. doi: 10.2196/56522 (PMC11612598; doi:10.2196/56522)

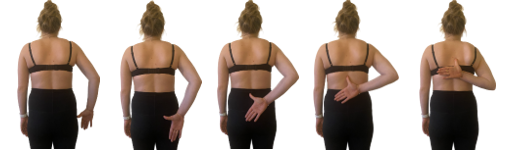

Supplement: Multimedia Appendix 2 [file resprot_v13i1e56522_app2.png]

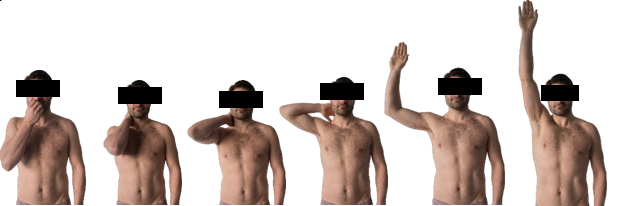

Supplement: Multimedia Appendix 3 [file resprot_v13i1e56522_app3.png]
